# Left circumflex artery pericardia fistula combined with huge pseudoaneurysm: a rare case report

**DOI:** 10.1186/s12872-021-02375-x

**Published:** 2021-11-27

**Authors:** Zhiyan Shen, Kun Xia, Xinfeng Liu, Rongpin Wang

**Affiliations:** 1 Department of Graduate School, Zunyi Medical University, Zunyi, 563000 China; 2 Department of Radiology, International Exemplary Cooperation Base of Precision Imaging for Diagnosis and Treatment, NHC Key Laboratory of Pulmonary Immune-related Diseases, Guizhou Provincial People’s Hospital, No. 83 Zhongshan East Road, Nan Ming District, Guiyang, 550002 Guizhou Province China

**Keywords:** Circumflex coronary artery, Coronary artery fistula, Pseudoaneurysm

## Abstract

**Background:**

Coronary artery fistula refers to an abnormal communication between a coronary artery and great vessel, a cardiac chamber or other structure. The left circumflex artery (LCX) pericardia fistula combined with huge pseudoaneurysm is extremely rare.

**Case presentation:**

A 39-year-old young female was admitted into our hospital because of palpitation and shortness of breath. Coronary computed tomography angiography (CCTA) showed a huge pseudoaneurysm located in pericardium. Coronary angiography revealed the LCX pericardia fistula. Then surgical treatment was performed. She was in good condition without complications after surgery.

**Conclusions:**

Coronary artery fistula combined with pseudoaneurysm can be caused by congenital factors. Early surgical treatment can relieve the patient's symptoms and prevent the occurrence of adverse cardiovascular events.

## Background

Coronary artery fistula (CAF) refers to an abnormal communication between a coronary artery and great vessel, a cardiac chamber or other structure [1]. It is rare with an incidence of 0.002% in the general population but the prevalence is 0.05–0.25% in patients who undergo coronary angiography [2]. Herein, we described a rare case of young female with both the LCX pericardia fistula and a huge pseudoaneurysm. She was treated successfully by surgery.

## Case presentation

A 39-year-old young female with no history of heart disease was admitted with palpitation, shortness of breath for 2 years and aggravation for 6 months. On admission, her blood pressure was 110/60 mmHg and her heart rate was 68 beats/min without cardiac murmurs. Her lower limb were not swollen. Electrocardiogram and laboratory examinations were normal range.

**Fig. 1:**
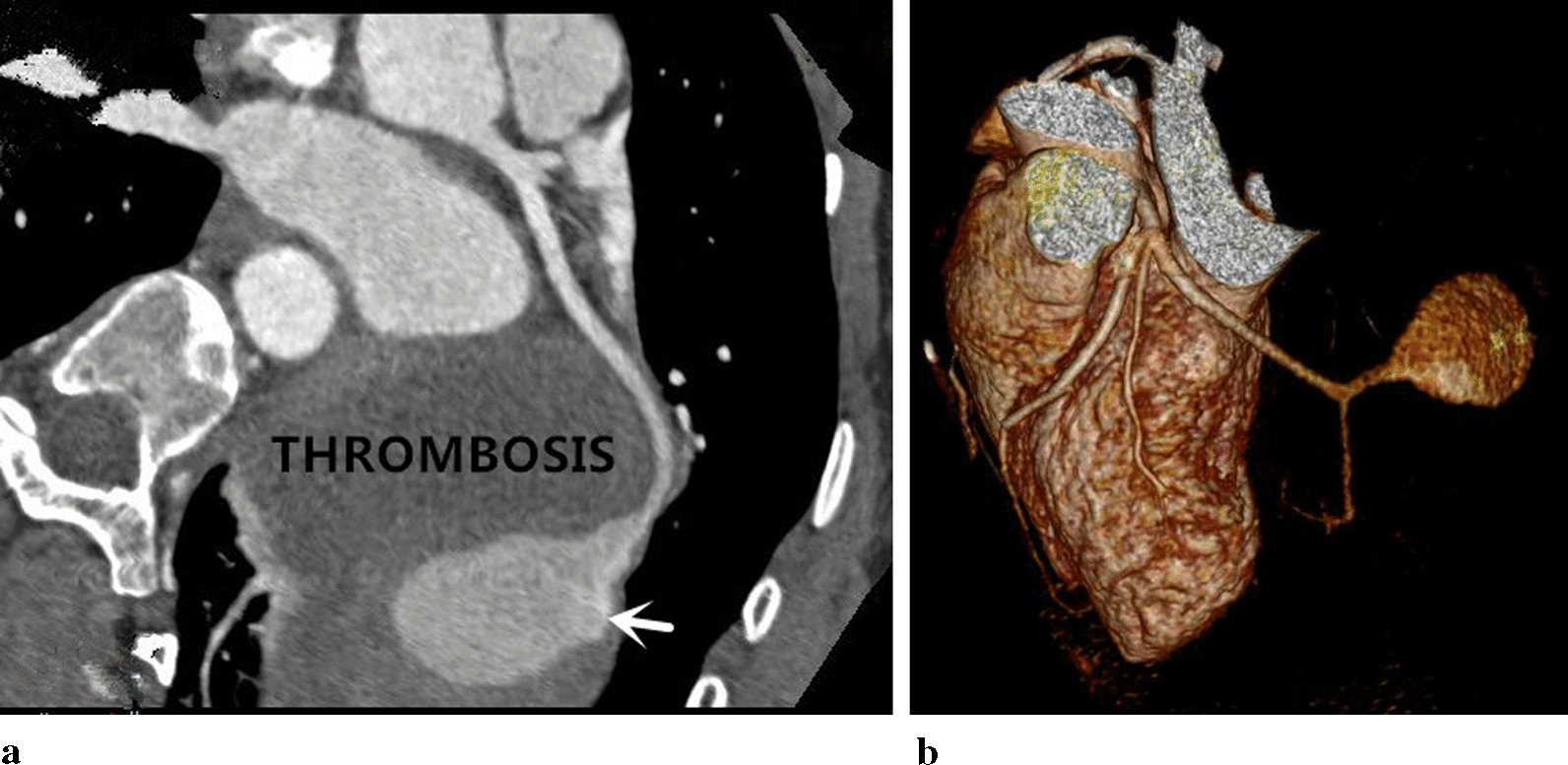
**a** Coronary computed tomography angiography (CCTA) revealed the giant LCX aneurysm with contrast agent filling observed in the lumen (white arrow) which contained huge intra-aneurysmal thrombosis. **b** Three-dimensional reconstruction showed the LCX distal pseudoaneurysm

**Fig. 2:**
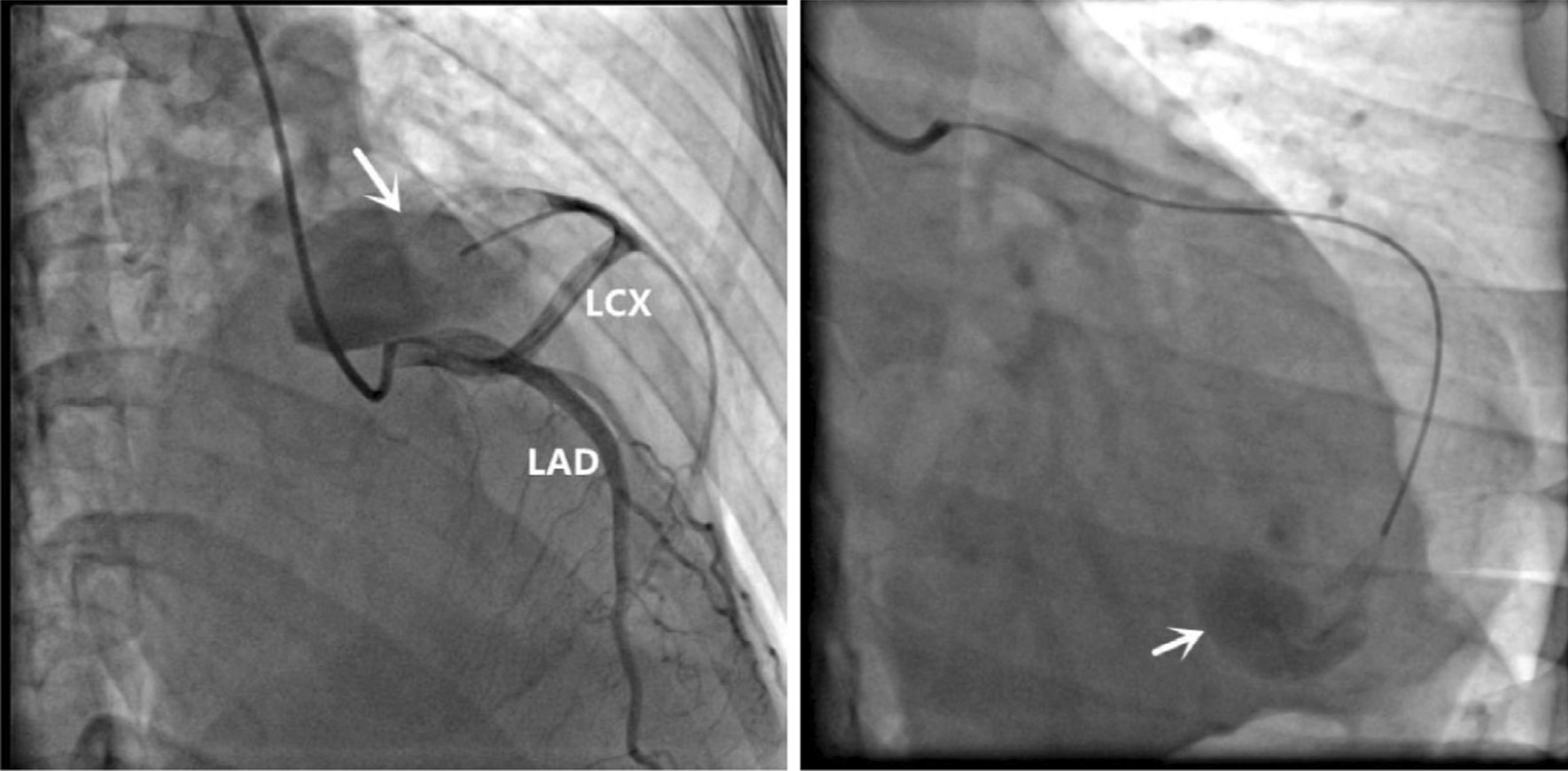
Selective left coronary artery angiography demonstrating the LCX fistula to the pericardium (white arrow)

Chest radiography revealed that her cardiac shadow was enlarged to the left. Echocardiography showed a mass of the pericardium. Coronary computed tomography angiography (CCTA) demonstrated that the distal segment of the LCX was significantly dilated with contrast agent filling and with huge thrombi (Fig. 1a) in the pericardium. Volumetic reconstruction showed that distal segment of the LCX were remarkably dilated (Fig. 1b). The left anterior descending branch and left coronary trunk were normal. The patient underwent the coronary angiography and selective micro catheter angiography of the LCX, which revealed a distal fistula of the LCX communicating with the pericardium (Fig. 2). The right coronary artery and the left ventriculography were normal. Therefore, a diagnosis of the LCX fistula complicated with false aneurysm was confirmed.

**Fig. 3:**
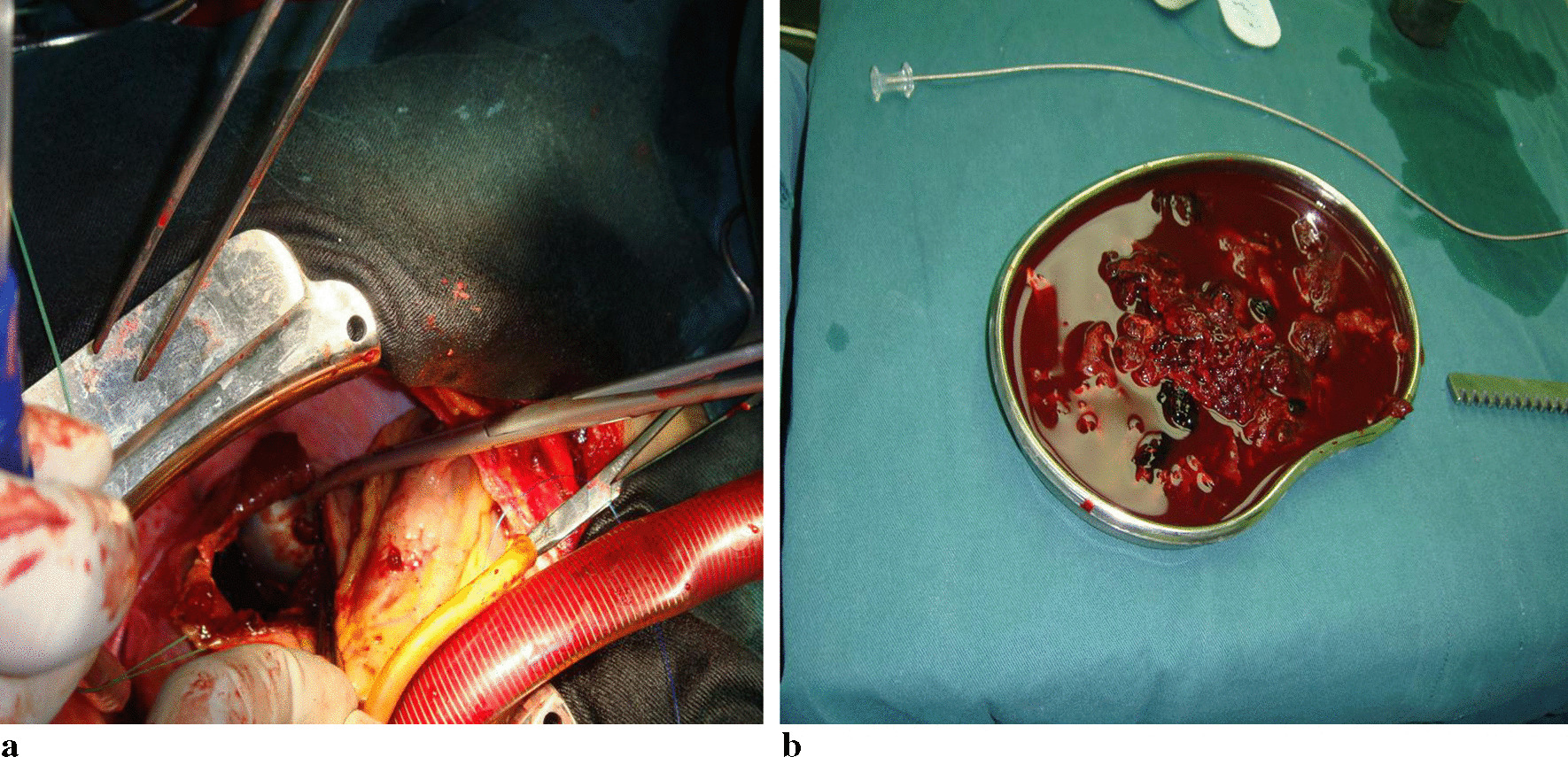
**a** Surgical view of many old thrombi inside the mass which was located under the epicardium. **b** Photograph of intra-pseudoaneury small thrombosis after surgery

The patient was admitted to the department of cardiac surgery for the LCX repair, partial resection of pseudo aneurysm wall and for coronary aneurysm thrombectomy after all examinations were complete. During the operation, a mass with a size about 25 × 20 mm was found in the left rear of the left ventricle, which was located under the epicardium. Then cardiopulmonary bypass was established, a massive thrombi inside the pseudoaneurysm were observed after the left rear of the mass was opened (Fig. 3a). And the total amount of thrombi was about 250 g (Fig. 3b). After that, the CAF was explored. It was found that the CAF opened on the outer wall of the pseudoaneurysm, with a length of about 3 mm. Finally, the fistula was sutured with 4–0 silk thread and most of the pseudoaneurysm wall was resected. The excised lesions were sent for pathological examination. The pathological diagnosis was pseudoaneurysm and mixed thrombi. Follow-up has been carried out for six years, the patient was in good condition after surgery.

## Discussion and conclusions

Most of the CAFs originate from the right coronary (52%), arise from the left anterior descending (30%), date from the left circumflex branch (18%), and over 90% of CAFs flow into the right heart [2]. The LCX fistula is very uncommon. The early embryonic development of infection and genetic factors may cause congenital heart disease, which is the primary reason of CAF [1]. Furthermore, coronary atherosclerosis, Takayasu's arteritis or trauma can lead to CAF [3]. In this case, the young patient had no history of coronary artery disease, vasculitis and trauma, which indicated most likely a congenial coronary fistula. Patients with CAF can have different symptoms, such as angina, congestive heart failure, bacterial endocarditis, cardiac arrhythmia or fistula rupture [4]. Although most patients remain commonly asymptomatic for life, CAF can enlarge and rupture [5]. The complications of CAF include myocardial ischaemia, thrombosis and embolism, cardiac failure, atrial fibrillation, rupture, endocarditis/endarteritis and arrhythmias [6]. In our case, the patient showed palpitation and shortness of breath, which is probably due to the contained rupture of the LCX pericardial fistula, followed by the formation of pseudoaneurysm and thrombosis. As the pseudoaneurysm continues to expand, it compresses the surrounding blood vessels, leading to poor reflux, secondary thrombosis and myocardial ischemia. It has also been reported that spontaneous rupture of aneurysmal fistula can cause haemopericardium [6]. Without closure of fistula, this patient could be at risk of pseudoaneurysm rupture and cardiac tamponade.

Conservative treatment, medication, transcatheter intervention and surgery are used to treat CAF [1]. Surgical closure of the CAF was preferred to treat female patients with a fistula that arises from the LCX and ends with a saccular aneurysm are at high risk of rupture, as reported by Said et al. [7], and the perioperative mortality reported with a surgical approach is 2–4% [4]. The possibility of rupture, a large shunt ratio, and progressive dilatation of the coronary fistulous aneurysm needs early resection of the aneurysm and closure of the fistula [8]. In order to alleviate the symptoms and prevent the rupture of pseudoaneurysm, the patient's fistula was repaired and resected most false aneurysm wall by surgery. So far, the patient has not had any cardiovascular adverse events after the operation.

In conclusion, the huge pseudoaneurysm due to the rupture of left circumflex artery pericardia fistula is extremely rare. Early surgery can reduce the risk of cardiac tamponade because of the continuous fistula and dilated pseudoaneurysm.

## Data Availability

The datasets used and/or analysed during the current study are available from the corresponding author on reasonable request.

## Abbreviations

CAF: Coronary artery fistula
CCTA: Coronary computed tomography angiography
LCX: Left circumflex artery
